# Osteonecrosis of the jaw induced by immune checkpoint inhibitors: an urgent need for attention

**DOI:** 10.1186/s12967-023-04482-z

**Published:** 2023-09-05

**Authors:** Xinya Li, Zaiwei Song, Rongsheng Zhao

**Affiliations:** 1 Department of Pharmacy, Peking University Third Hospital, 49 North Garden Road, Haidian District, Beijing, 100191 China; 2 Institute for Drug Evaluation, Peking University Health Science Center, Beijing, 100191 China; 3 Department of Pharmacy Administration and Clinical Pharmacy, School of Pharmaceutical Sciences, Peking University, Beijing, 100191 China

**Letter to the editor**

Since the approval of ipilimumab by the Food and Drug Administration (FDA) in 2011, immune checkpoint inhibitors (ICIs) have revolutionized the therapeutic landscape for multiple cancer types. However, immune-related adverse events (irAEs), which are unique to ICIs, impose a substantial burden on patients. Although whole-process monitoring and management strategies exist for various irAEs in current clinical guidelines and practice, a noticeable dearth of focus persists on ICI-related osteonecrosis of the jaw (ONJ). Medication-related ONJ typically presents as recurrent jaw infections, accompanied by soft tissue swelling and pain, often culminating in jaw necrosis and exposure, or even pathological fractures in severe cases [1]. Patients with ICI-related ONJ frequently necessitate the discontinuation or alteration of immunotherapy regimens, adversely impacting treatment efficacy and prognosis. This study endeavors to shed light on ICI-related ONJ by conducting an extensive query of the FDA Adverse Event Reporting System (FAERS) database and a review of case reports to draw attention to this concern and enhance clinical safety in using ICIs.

The FAERS, a pivotal component of the FDA's post-marketing safety surveillance initiative, compiles individual case safety reports (ICSRs) globally. We collected the raw data from the publicly accessible data platform, OpenVigil 2.1 [2], specifically targeting 22 ICIs. In reference to the Medical Dictionary for Regulatory Activities, we identified ICSRs of “osteonecrosis of jaw” from the initial FDA approval date of ICIs to March 31, 2023. Our disproportionality analysis used the proportional reporting ratio (PRR) with 95% confidence intervals and Chi-square statistic to compare specific drugs with others in the database. According to the criteria delineated by Evans et al. in 2001 [3], combinations of drug and ONJ meeting the following thresholds—with the number of reports > 2, a Chi-square statistic > 4, and a PRR > 2 were considered probably related.

We also conducted a comprehensive literature search in PubMed, Embase, the Cochrane Library, and Chinese databases up until May 2023, retrieving reports about ICI-related ONJ. This systematic exploration employed a set of keywords that were closely aligned with ICIs and ONJ. Reports were considered eligible for inclusion if they provided comprehensive patient information and offered insights into the onset and progression of ONJ.

In our exploration of OpenVigil 2.1, we identified 41 reports, which were distributed as atezolizumab (n = 5), ipilimumab (n = 5), nivolumab (n = 26), and pembrolizumab (n = 5). Among them, male patients (n = 16) were more represented than their female counterparts (n = 6). Of 20 reports with age information, ages ranged from 22 to 76 years. The most frequently reported indications were metastatic renal cell carcinoma (n = 7) and non-small cell lung cancer (n = 5) (Fig. 1). Our data analysis did not reveal statistically significant signals (Fig. 2).

**Fig. 1:**
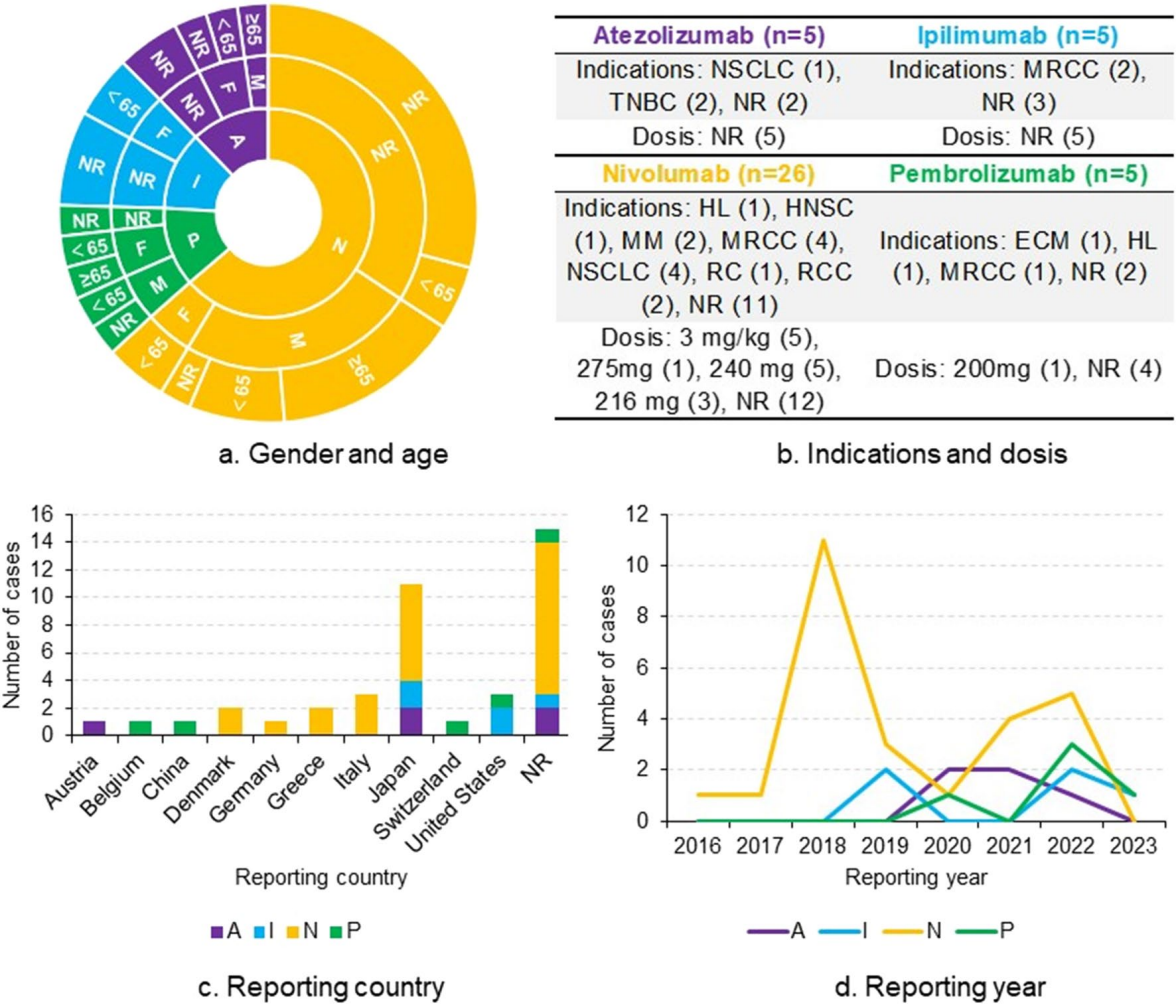
Descriptive characteristics of patients with osteonecrosis of the jaw induced by immune checkpoint inhibitors based on reports submitted to FAERS database. Note: A, atezolizumab; ECM, endometrial cancer metastatic; F, female; FAERS, the Food and Drug Administration's Adverse Event Reporting System; HL, Hodgkin's lymphoma; HNSC, head and neck squamous cell carcinoma; I, ipilimumab; kg, kilogram; M, male; mg, milligram; MM, malignant melanoma; MRCC, metastatic renal cell carcinoma; N, nivolumab; NSCLC, non-small cell lung cancer; NR, not reported; NA, not applicable; P, pembrolizumab; RC, renal cancer; RCC, renal cell carcinoma; TNBC, triple negative breast cancer

**Fig. 2:**
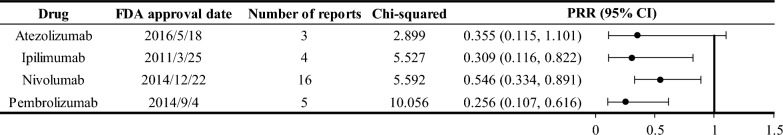
Association between immune checkpoint inhibitors and osteonecrosis of the jaw. CI, confidence intervals; FDA, Food and Drug Administration; PRR, proportional reporting ratio; The disproportionality analysis in the OpenVigil 2.1 used cleansed data from FDA Adverse Event Reporting System, by excluding reports with incomplete information

Our literature search yielded two pertinent case reports, each detailing instances of ONJ induced by ipilimumab and nivolumab, respectively [4, 5]. These cases shared some commonalities: both patients were male residents of the United States, and they both received ICIs for treating metastatic melanomas. The administration doses were tailored to their body weights. The onset of the first ONJ-related symptom appeared within 7 and 27 days after the initial dose, with recovery after 4 and 7 months of treatment. In the treatment of ONJ, both patients received amoxicillin-clavulanate. Additionally, one patient was administered ibuprofen, morphine, and prednisone as part of their therapeutic regimen, culminating in the necessity for a total mandibulectomy (Additional file 1: Table S1).

To the best of our knowledge, this study represents the inaugural comprehensive examination and presentation of ICI-related ONJ in the FAERS database. To date, the underlying pathophysiology of medication-related ONJ have not yet been fully elucidated. It is postulated that ICIs may cause ONJ by disrupting immune homeostasis. For instance, ipilimumab potentially elevates the systemic presence of activated T-cells [6]. Several factors may increase the risk of ICI-related ONJ. Firstly, ONJ pathogenesis involves pre-existing oral diseases and bacterial infections [7]. In one case, histopathologic evaluation reported a sequestrum containing bacterial colonies [5]. Furthermore, the combination of some high-risk drugs with ICIs may increase the incidence of ONJ. Case reports have documented ONJ occurrences in cancer patients receiving targeted therapies, specifically tyrosine kinase inhibitors and monoclonal antibodies [8, 9]. Moreover, the duration of bisphosphonate or antiresorptive therapy has been identified as a risk factor for the development of ONJ [10].

Our study has several limitations. Firstly, FAERS is a spontaneous reporting system characterized by reporting bias and missing data. Therefore, the data analysis results necessitate cautious interpretation. Secondly, ICI-related ONJ is relatively rare, resulting in a limited pool of available cases and studies. Given this scarcity, further monitoring and studies are needed to gain deeper insights.

In conclusion, heightened vigilance among healthcare professionals and patients regarding ICI-related ONJ is crucial, especially in the presence of risk factors. Comprehensive oral examinations should be standard practice before and during ICI treatment. Furthermore, multidisciplinary collaboration is essential for preventing and managing ICI-related ONJ.

### Supplementary Information

**Additional file 1: Table S1.** Characteristics of patients with osteonecrosis of the jaw induced by immune checkpoint inhibitors from case reports.

## Data Availability

All data generated or analyzed was included in this published letter.

## Abbreviations

FDA: Food and drug administration
ICIs: Immune checkpoint inhibitors
irAEs: Immune-related adverse events
ONJ: Osteonecrosis of the jaw
FAERS: FDA adverse event reporting system
ICSRs: Individual case safety reports
PRR: Proportional reporting ratio
