# Supplementary material for: Osteonecrosis of the jaw induced by immune checkpoint inhibitors: an urgent need for attention
Source: J Transl Med. 2023 Sep 5;21:592. doi: 10.1186/s12967-023-04482-z (PMC10478485; doi:10.1186/s12967-023-04482-z)
Supplement: Supplementary file 1 — Additional file 1: Table S1. Characteristics of patients with osteonecrosis of the jaw induced by immune checkpoint inhibitors from case reports. [file 12967_2023_4482_MOESM1_ESM.docx]

**Additional material**

**Additional Table S1 Characteristics of patients with osteonecrosis of the jaw induced by immune checkpoint inhibitors from case reports**

| Study ID | Owosho 2015 | Pundole 2020 |
| --- | --- | --- |
| Publication country | United States | United States |
| Publication type | Letter to the Editor | Case report |
| Age | 52 | 75 |
| Gender | Male | Male |
| Pre-existing diseases | Not reported | Chronic obstructive pulmonary disease, hypertension |
| Previous medications | No anti-resorptive medications | Not reported |
| Indications | Metastatic melanoma of unknown primary, stage IV with metastases to the left iliac region as well as pancreatic head; diagnosed in early 2014 | Metastatic melanoma |
| Radiotherapy | Palliative radiotherapy, 27 Gy in 3 fractions completed in March 2014 | No |
| Suspected drugs | Ipilimumab | Nivolumab |
| Administration regimen | 230 mg every 3 weeks for a total of 4 doses | 240 mg every 2 weeks |
| Time of initial immunotherapy | March 2014 | Early March 2018 |
| Onset time of first symptom | Six days after the second dose | One week after the first dose |
| Duration of osteonecrosis of the jaw | About 4 months | About 8 months |
| Treatment for osteonecrosis of the jaw | Irrigated with 0.12% chlorhexidine 3 times per day, Augmentin 875 mg bid for 7 days | Oral amoxicillin-clavulanate, prednisone 40 mg daily for 23 days, augmentin-clavulanate (875-125 mg bid x 14 days), total mandibulectomy |
| Immunotherapy regimen adjustment | Not reported | Discontinued treatment, an additional three cycles of 240 mg of nivolumab q2 weeks, then switched to an every 3-week schedule for cycles 7 and 8 |
